# Tuberculous Otitis Media with Facial Paralysis: A Clinical and Microbiological Diagnosis—A Case Report

**DOI:** 10.1155/2011/932608

**Published:** 2011-07-02

**Authors:** Nicola Quaranta, Paolo Petrone, Alexandra Michailidou, Luisa Miragliotta, Marilina Santantonio, Raffaele Del Prete, Adriana Mosca, Giuseppe Miragliotta

**Affiliations:** ^1^Otorhinolaryngology clinic “G. Lugli”, Otologic and Neurotologic Surgery, University of Bari, 70124 Bari, Italy; ^2^Section of Microbiology, Department MIDIM, University of Bari, Policlinico, Piazza Giulio Cesare, 70124 Bari, Italy

## Abstract

The tuberculosis of the ear is rare, and in most cases the clinical picture resembles that of a chronic otitis media. The diagnosis is often delayed, and this can lead to irreversible complications such as hearing loss and/or facial paralysis. In view of its rare occurrence, we report a case of primary tuberculous otitis media in a 87-year-old female patient. The diagnosis was made on the basis of both histological and microbiological findings. In particular, gene amplification techniques such as real-time polymerase chain reaction are useful method for rapid diagnosis and detecting tuberculous bacilli usually present at very low number. Early diagnosis is essential for the prompt institution of antituberculous therapy.

## 1. Introduction

Tuberculosis (TB) is a serious and contagious infectious disease potentially affecting various organs and tissues. Tuberculous otitis media (TOM) is an uncommon, insidious, and frequently misdiagnosed form of TB [1]. In particular, TOM is usually secondary to direct transmission from adjacent organs (i.e., the lungs, larynx, pharynx, and nose), while the primary form has been rarely reported. Half of the cases have no other evidence of present or past infection, and its diagnosis is often delayed due to the rarity of this disease or its usually indolent course [2, 3]. 

We present a case of TOM in a patient who had an atypical presentation and no previous history of TB, whose diagnosis has been made possible through histological and molecular biology techniques.

## 2. Case Report

A 87-year-old woman with a history of pulmonary fibrosis, chronic heart failure, and hepatitis C infection was admitted at our hospital in April 2010 with right ear otorrhea, otalgia, tinnitus, and peripheral facial nerve paralysis. The patient reported that symptoms gradually appeared over the last two years and for this reason in May 2009 she underwent mastoid curettage with concho-meatoplasty in local anaesthesia due to the high clinical risk of general anaesthesia at another institution. At that stage no serologic, histologic, or microbiologic analysis was performed. 

**Figure 1:**
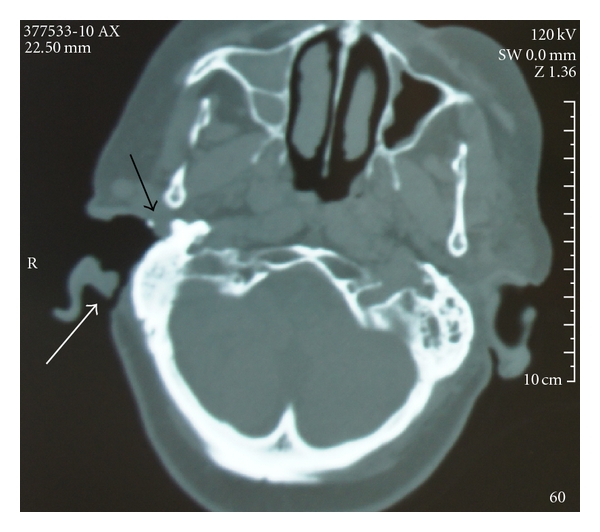
Axial high-resolution CT scan showing the retroauricular fistula (white arrow), sclerotic mastoid and granulation tissue in the middle ear area (black arrow).

On admission the patient was apyretic, even if tachycardic (heart rate 98/min). Blood pressure was 110/75 mmHg and oxygenation saturation 98%. Examination of the right ear showed wide conchomeatoplasty, a large (5 cm) postauricular cutaneous mastoid fistula in the region of the previous retroauricular incision. The mastoid bone was partly uncovered and partly lined by granulation tissue that extended in the middle ear region with no tympanic membrane. Periauricular cutaneous lesions were also evident. Facial nerve function was House Brackmann grade III. Left ear was normal as well as the rest of the otolaryngologic examination. Hearing test showed a right dead ear and severe sensorineural hearing loss on the left side. Laboratory values included a white blood cell count of 9.530/mm^3^ (86.5% segmented neutrophils, 7.9 lymphocytes, 5% monocytes, 0.5% eosinophils, and 0.1% basophils), haemoglobin 13.0 g/dL, and platelets 185.000/mm^3^. Blood and urine cultures as well as sputum examination were negative. The chest X-ray was normal while high resolution computed tomographic (CT) scan of the lung showed a reticular pattern on all the pulmonary areas, with associated fibrotic areas. CT scan of temporal bones showed the signs of the previous surgery, as well as inflammatory tissue in the right external auditory canal, the middle ear and mastoid (Figure 1). 

**Figure 2:**
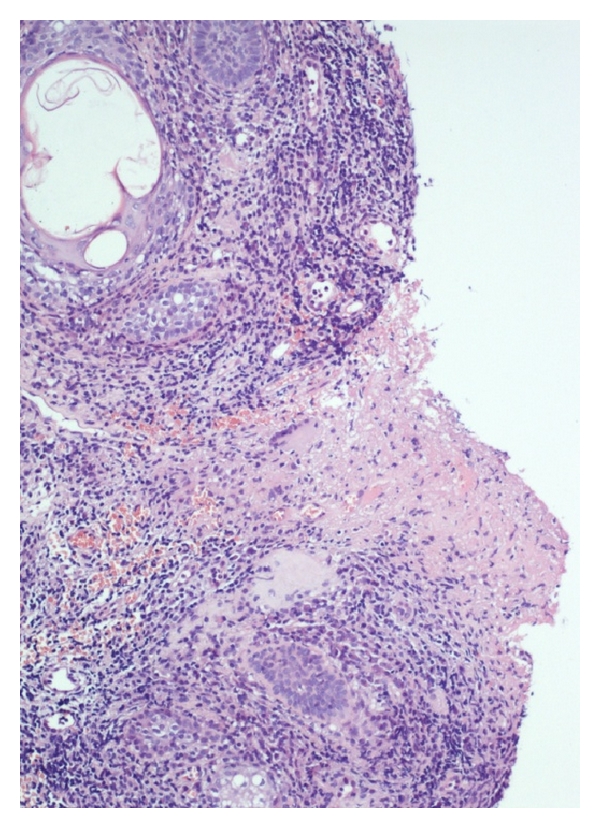
Granulomatous inflammation, Langhans cells, and caseation necrosis (100x).

These findings along with chest X-ray and HRT-CT lung scan, negative for TBC, allowed to exclude a secondary TBC form. The histological examination of the granulation tissue showed caseous necrosis and specific granulation with epithelioid cells and Langhans type giant cells (Figure 2). 

A swab of the secretion revealed no alcohol-acid-resistant bacilli when Ziehl Neelsen staining method was performed, while the real-time-polymerase chain reaction (rt-PCR) allowed the detection of *Mycobacterium tuberculosis*. On the basis of the laboratory findings anti-TB treatment including rifampicin and isoniazid was started together with local medications of the mastoid cavity with rifampicin drops. On follow-up facial nerve paralysis resolved after the second month of therapy, and the conditions of the mastoid cavity progressively improved up to the point that the retroauricular fistula was successfully sutured and the granulation tissue disappeared.

## 3. Discussion

TOM is a rare cause of chronic suppurative infection of the middle ear, ranging from 0.05 to 0.9% of the chronic otitis media TB [3]. Because of similar symptoms and signs its differential diagnosis from non-TB chronic otitis media may be quite difficult TB [4]. In addition, the clinical manifestations of this disease are not always in accordance with the description of the classical triad of painless otorrhea, multiple tympanic perforations, and facial palsy TB [5, 6]. Our case presented in an old patient whereas it is generally accepted that TOM has a relatively high prevalence among children [3] and an associated peripheral facial paralysis has been described in 15% to 40% cases, mostly children TB [3, 5]. In adults TOM is more commonly seen in non-immunocompetent patients. However, in immunocompetent individuals a considerable delay before the diagnosis is possible because of low prevalence of the disease and insidious clinical signs. In the present case the diagnosis of TOM was made more than 1 year after the presentation of symptoms. In fact, although the patient presented with chronic discharge, ear pain, and facial paralysis, no attempt to reveal the aetiology of the disease was done. On the contrary, mastoid curettage and conchomeatoplasty resulted in the perseverance of the disease and in the occurrence of a large retroauricular fistula in the region of the incision. According to most authors who report that histological findings, such as granulomas, Langhans giant cells, and caseation necrosis, in association with biomolecular positivity (PCR), represent the cardinal diagnostic elements of TOM [6, 7], our diagnosis was formulated and confirmed by the above two diagnostic tool. Whereas PCR amplification can be used to increase diagnostic accuracy, Ziehl-Nielsen staining should not be considered as a sensitive test, since tuberculous lesions of the ear show low bacterial concentrations [8] which further decline with the use of antibiotic ear drops (e.g., aminoglycoside) [9]. While the diverse signs and symptoms observed confirm that TOM cannot be definitively diagnosed on the basis of clinical manifestations alone, definitive identification of mycobacteria is not always easy to achieve. In this regard, pathologic findings are increasingly taking an important place in the diagnosis since biomolecular techniques are now available and can play an important role in diagnosing tuberculosis of the ear as early as possible to avoid unnecessary treatments and sequelae. All authors agree that treatment of TOM, including the non-lung-involving forms, has to include anti-TBC pharmacological protocols for at least six months [10]. With regard to the role of surgical treatment, it should be considered for the treatment of complications, such as subperiosteal abscesses, but it is controversial in case of noncomplicated TOM [6].

## References

[1] Odetoyinbo O (1988). Early diagnosis of tuberculous otitis media. *Journal of Laryngology and Otology*.

[2] Vita V, Printza A, Zaraboukas T (2002). Tuberculous otitis media: a difficult diagnosis and report of four cases. *Pathology Research and Practice*.

[3] Vaamonde P, Castro C, García-Soto N, Labella T, Lozano A (2004). Tuberculous otitis media: a significant diagnostic challenge. *Otolaryngology*.

[4] Awan MS, Salahuddin I (2002). Tuberculous otitis media: two case reports and literature review. *Ear, Nose and Throat Journal*.

[5] Skolnik PR, Nadol JB, Baker AS (1986). Tuberculosis of the middle ear: review of the literature with an instructive case report. *Reviews of Infectious Diseases*.

[6] Cho YS, Lee HS, Kim SW (2006). Tuberculous otitis media: a clinical and radiologic analysis of 52 patients. *Laryngoscope*.

[7] Bhalla RK, Jones TM, Rothburn MM, Swift AC (2001). Tuberculous otitis media—a diagnostic dilemma. *Auris Nasus Larynx*.

[8] Lee PYC, Drysdale AJ (1993). Tuberculous otitis media: a difficult diagnosis. *Journal of Laryngology and Otology*.

[9] De Paep K, Offeciers FE, Van de Heyning P, Claes J, Marquet J (1989). Tuberculosis in the middle ear: 5 case reports. *Acta Oto-Rhino-Laryngologica Belgica*.

[10] Dunlap NE, Bass J, Fujiwara P, Hopewell P, Horsburgh CR, Salfinger M (2000). Diagnostic standards and classification of tuberculosis in adults and children. *American Journal of Respiratory and Critical Care Medicine*.

